# Presence of Heavy Metals in Irrigation Water, Soils, Fruits, and Vegetables: Health Risk Assessment in Peri-Urban Boumerdes City, Algeria

**DOI:** 10.3390/molecules29174187

**Published:** 2024-09-04

**Authors:** Mohamed Younes Aksouh, Naima Boudieb, Nadjib Benosmane, Yacine Moussaoui, Rajmund Michalski, Justyna Klyta, Joanna Kończyk

**Affiliations:** 1 Laboratory of Treatment and Shape of Polymers, University M’Hamed Bougara of Boumerdes, Boumerdes 35000, Algeria; 2 Department of Chemistry, Faculty of Science, University M’hamed Bougara of Boumerdes, Boumerdes 35000, Algeria; 3 Laboratory of Heterocyclic Compounds, Faculty of Chemistry, University Houari, Boumediene of Science and Technology, Bab Ezzouar 16111, Algeria; 4 Laboratoire de Valorisation et Promotion des Ressources Sahariennes (VPRS), Faculté des Mathématiques et Sciences de la Matière, Université Kasdi Merbah (UKMO), Ouargla 30000, Algeria; 5 Laboratoire des Sciences et Techniques de l’Environnement, Ecole Nationale Polytechnique, El Harrach 16200, Algeria; 6 Institute of Environmental Engineering of Polish Academy of Sciences, 41-819 Zabrze, Poland; rajmund.michalski@ipispan.edu.pl (R.M.);; 7 Institute of Chemistry, Faculty of Science & Technology, Jan Dlugosz University in Czestochowa, 13/15 Armii Krajowej Avenue, 42-200 Czestochowa, Poland

**Keywords:** heavy metals, irrigation water, soil, fruit, vegetables, food, health risk, Boumerdes

## Abstract

This study investigates heavy metal contamination in soils, irrigation water, and agricultural produce (fruits: *Vitis vinifera* (grape), *Cucumis melo var. saccharimus* (melon), and *Citrullus vulgaris. Schrade* (watermelon); vegetables: *Lycopersicum esculentum* L. (tomato), *Cucurbita pepo* (zucchini), *Daucus carota* (carrot), *Lactuca sativa* (lettuce), *Convolvulus Batatas* (potato), and *Capsicum annuum* L. (green pepper)) in the Boumerdes region of Algeria. The concentrations of seven heavy metals (cadmium (Cd), chromium (Cr), copper (Cu), iron (Fe), nickel (Ni), lead (Pb), and zinc (Zn)) in soil and food samples were analyzed using atomic absorption spectrometry. Health risks associated with these metals were evaluated through the estimated daily intake (EDI), non-carcinogenic risks (using target hazard quotient (THQ), total target hazard quotient (TTHQ), and hazard index (HI)), and carcinogenic risks (cancer risk factor (CR)). Statistical analyses, including cluster analysis (CA) and Pearson correlation, were conducted to interpret the data. The results revealed the highest metal transfer as follows: Cd was most significantly transferred to tomatoes and watermelons; Cr to carrots; Cu to tomatoes; and Fe, Ni, Pb, and Zn to lettuce. Among fruits, the highest EDI values were for Zn (2.54·10^−3^ mg/day) and Cu (1.17·10^−3^ mg/day), with melons showing the highest Zn levels. For vegetables, the highest EDI values were for Fe (1.68·10^−2^ mg/day) and Zn (8.37·10^−3^ mg/day), with potatoes showing the highest Fe levels. Although all heavy metal concentrations were within the World Health Organization’s permissible limits, the HI and TTHQ values indicated potential health risks, particularly from vegetable consumption. These findings suggest the need for ongoing monitoring to ensure food safety and mitigate health risks associated with heavy metal contamination.

## 1. Introduction

The contamination of the food chain by heavy metals (Cd, Cu, Ni, Pb, Zn, etc.) is considered one of the main environmental routes of human exposure leading to a potential health risk. The translocation of these metals in the food chain is one of the consequences of their presence in soils and environmental water [1,2,3,4]. Since heavy metals are non-biodegradable, persistent in a natural environment, and mobile, they can easily enter the human body with food and accumulate in different parts of the body, causing, even at low concentrations, adverse health effects [5,6,7,8]. Heavy metals such as cadmium and lead are one of the most toxic metals with carcinogenic and mutagenic activities [9,10]. Cadmium exposure can result in serious systemic diseases and damage to the skeletal and cardiovascular systems, the kidneys, and the liver, as well as problems with seeing and hearing [10,11]. Lead is a highly poisonous metal affecting many organ systems, especially the nervous, respiratory, digestive, and reproductive systems, and prevents enzymes from performing their normal activities. Continuous exposure of the body (e.g., ingestion and skin contact with highly contaminated soil and/or dust) leads to a decline in neurocognitive functions, nephrotoxicity, hypertension, decreased reproductivity, cataract formation, hearing defects, dental problems, and mucocutaneous signs [7,12,13,14]. In addition, the ingestion of heavy metals can seriously cause a depletion of certain essential nutrients in the body, which in turn causes a decrease in immunological defenses, intrauterine growth retardation, psychosocial dysfunctions, disabilities associated with malnutrition, and a high prevalence of upper gastrointestinal cancer [9]. It was found that kidney stone incidence increases with a high Pb exposure in males and with Pb and Cd co-exposure in males and females [15].

The presence of heavy metals in the environment may result from natural processes (e.g., weathering of metal-containing rocks, volcanic eruptions, forest fires) or human activities [8]. The main anthropogenic sources of heavy metals are mining, the metallurgical industry, fertilizers and pesticides applied in soil cultivation, medical waste, factory and car engine emissions, incinerators and ashes from waste incineration, municipal landfills, wastewater effluents, and sewage sludge [16,17]. When the metals are released from their sources, they enter the air, water, and soil and are transported into the environment together with aerosols, surface water and groundwater, suspended solids, or bottom sediments. Both the concentration of individual metals and their total concentration in each part of the environment are crucial to determine their impact on crops and the human organism. The availability of the metal to living organisms is mainly controlled by the dissolution of the carrier minerals and then by the adsorption and/or precipitation of this metal as a result of the geochemical reactions taking place in aerosol, water, or sediment [18,19]. The metals released in this way accumulate in plants grown in polluted areas, which enter the body of the consumers as food. Usually, fruits and vegetables are considered the healthiest for the consumer’s body; however, their cultivation place and method determine whether they provide the body with the proper ingredients [3,20].

This study aimed to determine the potential health risks of consuming popular fruits (grape, melon, and watermelon) and vegetables (tomato, zucchini, carrot, lettuce, potato, and green pepper) grown in four Algerian peri-urban regions (Boudouaou, Corso, Dellys, and Naceria) localized near Boumerdes city. This area is one of the most important areas in the production of agricultural products in Algeria. In order to compensate for the hydric shortages, the crops are irrigated with groundwater, river water, or treated sewage and wastewater from the municipal sewage treatment plant [21]. Both ground- and surface water are exposed to leachates from these landfills, and wastewater can be a potential source of hazardous elements in the crops. To achieve this goal, the concentrations of seven heavy metals (copper, zinc, iron, chromium, cadmium, nickel, and lead) in the river and groundwaters, soils, and fruits and vegetables were determined, and the transfer factor of each heavy metal from different agricultural activities was established. To date, few papers have described studies of the environmental quality in the Boumerdes region [21,22,23]; however, none of them focused on assessing the risk of heavy metals in the crops grown there. The knowledge of the heavy metal content in nationally representative samples of food produced in the Boumerdes region is extremely important in terms of the environmental effect on agriculture, the quality of crops, and consumers’ health.

## 2. Results and Discussion

### 2.1. Metal Concentration in Irrigation Water

The pH of the water used for the crop irrigation decreased in the order of Boudouaou (pH = 7.2) > Corso (pH = 7.0) > Naceria (pH = 6.7) > Dellys (pH = 5.5). The mean concentration values of heavy metals determined in the groundwater of the Boudouaou region and the river water of the Corso, Naceria, and Dellys regions and the maximum levels (MLs) recommended for irrigation water by The Food and Agriculture Organization of the United Nations and International Water Management Institute (FAO/IWMI) [24] and the United States Environmental Protection Agency (USEPA) [25] are presented in Table 1.

No carcinogenic cadmium was found in any tested waters in concentrations above the detection limit (LOD) of 0.5 µg/L. Chromium concentrations below the LOD of 3 µg/L were found only in river water applied in Corso and Naceria farmlands and one groundwater applied for zucchini irrigation in Boudouaou farmland. Other waters contained from 0.09 to 0.26 mg Cr/L, with values exceeding the ML for irrigation waters for carrot and green pepper cultivations. The concentration of copper in all tested waters was above the recommended value and ranged from 0.56 to 1.17 mg/L. The ML value was also exceeded for nickel concentration in the waters for the irrigation of zucchini (0.87 mg/L), potato (0.91 mg/L), and tomato (1.98 mg/L). The concentrations of Pb, Fe, and Zn were below the recommended value. The Pb content ranged from 0.03 to 0.82 mg/L, with noticeably higher values recorded in the water for the irrigation of lettuce and potato in the Dellys region (0.82 and 0.42 mg/L, respectively). The highest values were for water used to irrigate green peppers in the Dellys region (0.26 mg/L) and carrots in the Boudouaou region (0.19 mg/L). In turn, the concentrations of Fe and Ni changed from 0.04 (Naceria, watermelon irrigation) to 1.10 mg/L (Dellys, lettuce irrigation) and from 0.05 (Corso, grape irrigation) to 1.02 mg/L (Boudouaou, zucchini irrigation). Taking into account the total content of heavy metals analyzed (in mg/L), the purity of the water used to irrigate crops was the highest in the Naceria region (0.86) and decreased in the following series: Corso (2.35); Boudouaou (9.28); and Dellys (9.80).

**Figure 1:**
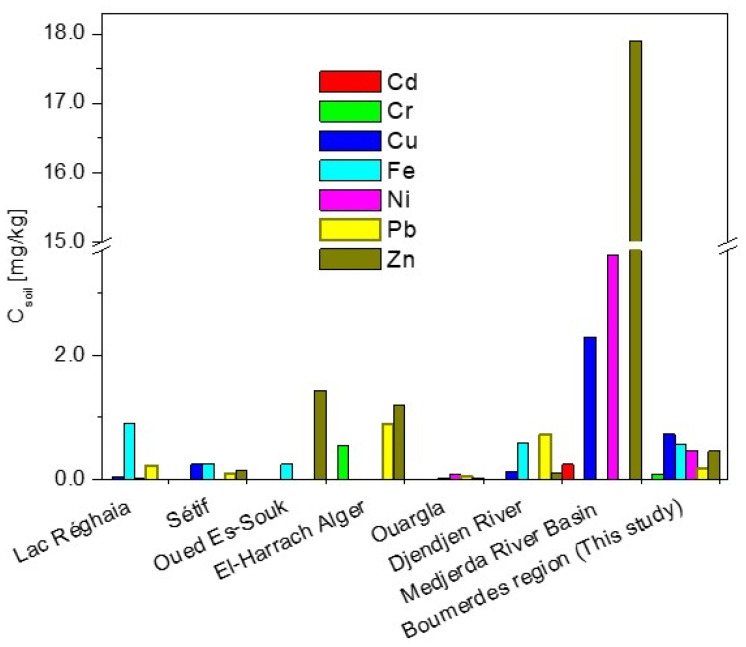
Comparison of the metal content in the waters of different regions of Algeria [26,27,28,29,30,31,32].

From the comparison of the obtained results with the literature data (Figure 1), it can be seen that the tested waters of the Boumerdes region used in crop irrigation definitely contain more copper, lead, and nickel than the waters of other regions of Algeria [26,27,28,29,30,31,32]. In turn, the iron content was comparable to the range of this element in the waters of Reghaia Lak [26] and several times lower than that in the waters in other regions [27,28,31]. Zinc concentrations were like those in the waters of Oued Es-Souk [28] and El Harrach River [29], except for Djendjen River [32], with several times higher concentrations of this element, and Ain Azel [33], with a zinc content of 30 mg/kg. The tested waters have a significantly lower chromium content (ca. 0.26 mg/L) compared to other Algerian waters but a higher iron content with 0.04 to 1.10 mg Fe per 1 L of water.

### 2.2. Metal Concentration in Soils

The pH and mean values of the metal concentrations in the studied soils are presented in Table 2. Soil pH is the main variable influencing biological, chemical, and physical properties and plant growth. It was found that even the smallest amount of acid causes a change in the pH, resulting in the increased mobility of metals in the soil environment [34]. The studied soils are weakly alkaline, from neutral to weakly acidic. The pH of 6.32 and 6.57 for the soils of the Dellys (lettuce cultivation) and Boudouaou regions (zucchini cultivation), respectively, may affect the mobility of heavy metals present in these soils.

The concentrations of heavy metals analyzed in the agricultural soils are below the maximum limits (MLs) authorized by the European Commission [35] and the State Administration of Market Regulation (for Fe) [36] (Table 2). Among the tested metals, significantly higher concentrations were recorded for Cu, Fe, Pb, and Zn than for Cd, Cr, and Ni. Cadmium was not found in amounts above the LOD in Dellys soils and Boudouaou soils with carrot cultivation. In the other soils, the concentration of this element ranged from 20 to 90 µg/kg. Chromium has been detected in amounts from 2.24 mg/kg in Boudouaou soil (carrot cultivation) to 12.17 mg/kg in Corso soil (melon cultivation); nickel—from 3.25 mg/kg in Boudouaou (carrot cultivation) to 21.25 mg/kg in Corso (grape cultivation); and lead—from 49.22 mg/kg in Naceria soil to 110.25 mg/kg in Dellys soil (potato cultivation). The Cu and Fe contents were the lowest in Dellys soil (potato cultivation; 38.25 and 42.74 mg/kg, respectively) and the highest in Boudouaou (zucchini cultivation; 78.00 mg/kg) and Dellys (potato cultivation; 120.42 mg/kg), respectively. The Zn concentration ranged from 70.12 mg/kg for Naceria soil to 108.21 mg/kg for Dellys soil (lettuce cultivation). Ghemmit-Doulache [23] determined the concentrations of these metals in 1 kg of soil irrigated by treated wastewater from the Treatment Station Treated Wastewater of Boumerdes at the levels of 0.0275 mg of Cd; 50.53 mg of Cr; 18.03 mg of Cu; 12.92 mg of Ni; 23.28 mg of Pb; and 69.65 mg of Zn.

For the determination of the correlation between the soil pH and heavy metal concentrations in the soils, Pearson coefficients were calculated and are listed in Table 3.

The soil pH does not correlate significantly with the studied metal concentration. In turn, Fe has a significant negative correlation with Cd, Cr, and Ni, indicating that an increase in the Fe concentration in the soil leads to a decrease in the Cd, Cr, and Ni concentrations. Additionally, a significant positive correlation between Cd, Cr, and Ni and Fe, Pb, and Zn has been observed, which suggests that these metal groups originated from similar anthropogenic sources. It is difficult to clearly indicate the significant impact of the industrial activities indicated in Section 3.1 on the quality of the tested soils; therefore, the reasons for the presence of heavy metals in these soils should be sought in municipal wastes and the pesticides and/or mineral fertilizers used. The bioavailability of Cd, Cu, Zn, and Pb present in the soil from the peri-urban region of New Delhi also showed a lack of relationship with the soil pH [37].

**Figure 2:**
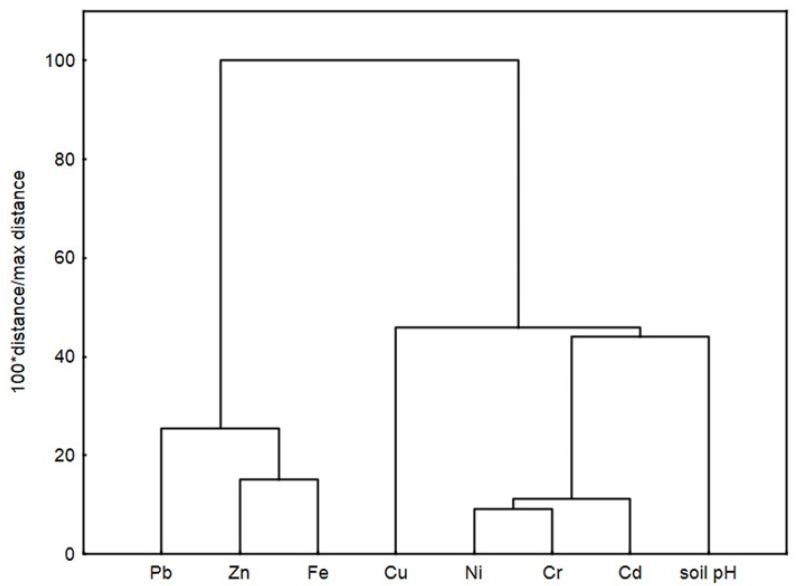
Dendrogram resulting from the hierarchical cluster analysis of the heavy metal concentrations in the studied soils.

As shown in Figure 2, the dendrogram resulting from the hierarchical cluster analysis (CA) indicates the presence of two main clusters with subclusters. The first cluster containing Pb, Zn, and Fe confirms a good correlation between these metals, with a closer relation between Zn and Fe. The second cluster containing Cd, Cr, Ni, Cu, and soil pH indicates a good correlation between Ni and Cr, a weaker correlation between this metal pair and Cd, and no correlation with Cu. In turn, the Cu concentration correlated well only with the pH of the soil.

**Figure 3:**
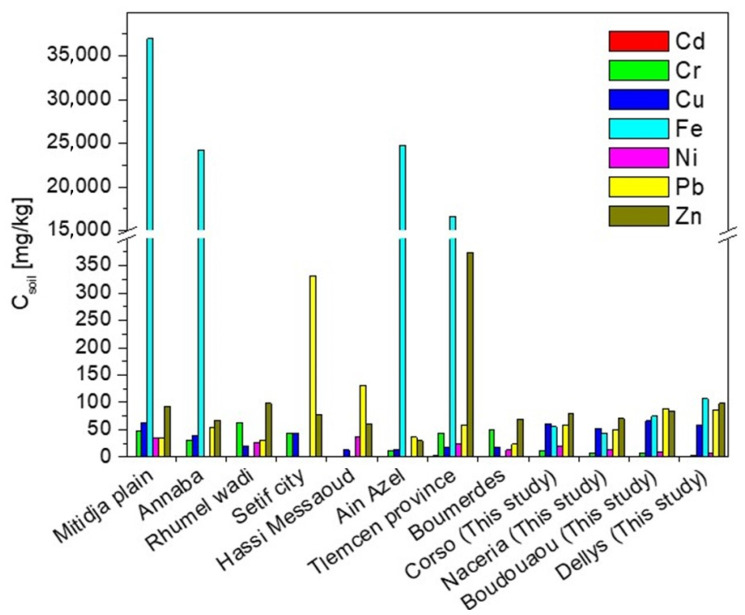
Comparison of the metal content in soils of different regions of Algeria [23,33,38,39,40,41,42,43].

When comparing the obtained results with the literature data (Figure 3), it can be concluded that the tested soils of all regions of Boumerdes contain less cadmium, chromium, and nickel but more lead than the soils of other regions of Algeria [33,38,39,40,41,42,43]. In turn, the copper content was comparable to the range of this element in the soils of the Mitidja plain [38], Annaba [39], and Setif city [41] and several times higher than in soils in other regions. The zinc concentrations were like those in soils in other regions, except for Tlemcen province [43], with four times higher concentrations of this element, and Ain Azel [33], with a zinc content of 30 mg/kg. The tested soils have a significantly lower iron content (ca. 80 mg/kg) compared to other Algerian soils, with 16.7 to 37 g Fe per 1 kg soil in the Tlemcen province [43] and Mitidja plain [38], respectively.

### 2.3. Metal Concentration in Fruits and Vegetables

The average concentrations of heavy metals found in fresh fruits and vegetables sampled in the studied area are summarized in Table 4.

Cadmium and chromium were not detected in the studied food in concentrations over the LOD value of 0.013 mg/kg, except in carrot, in which 0.090 mg of Cr per 1 kg was found. The contents of other metals varied as follows: from 0.94 to 13.01 mg/kg (in grape and tomato, respectively) for Cu; from 0.94 to 54.20 mg/kg (in grape and lettuce, respectively) for Fe; from <0.68 to 1.01 mg/kg (in grape, melon, zucchini, and green pepper and in lettuce, respectively) for Ni; from <0.42 to 3.45 mg/kg (in melon and watermelon and in lettuce, respectively) for Pb; and from 1.33 to 19.52 mg/kg (in grape and lettuce, respectively) for Zn. In most cases, the metal concentrations in the tested foods were below the maximum level (ML) recommended by the Joint World Health Organization and Food and Agriculture Organization of the United Nations/WHO Food [44], except lead, which was present in all tested fruits and vegetables in amounts exceeding the recommended safety limit of 0.1 mg/kg.

The content of the tested metals in individual crops differed depending on the plant and was lower in the case of fruits. The total content (in mg/kg) of all tested heavy metals in fruits and vegetables increased in the following order: watermelon ≅ grape < melon < zucchini < tomato < green pepper < carrot < potato < lettuce. The high content of heavy metals in lettuce is attributed to its physiology. The large leaf area and high rate of transpiration and plant growth may have a key impact on the ability to absorb heavy metals from water and soil and to accumulate them in the plant [45]. Rutigliano et al. [46] identified higher amounts of Cd, Cr, and Ni in lettuce *Lactuca sativa* than in pumpkin Cucurbita pep. grown in Southern Italy. The Pb content of the tested tomatoes (1.11 mg/kg) was slightly higher than that of tomatoes grown in North-East Algeria (Jijel, 0.82 mg/kg) [47] or Pakistan (0.98 mg/kg) [48] but higher than in Polish tomatoes (<0.004–0.022 mg/kg fresh mass) [49]. However, in the tested tomatoes and Polish tomatoes [49], no cadmium was detected in amounts above 0.047 mg/kg, while Bounar et al. [47] and De Sousa et al. [48] found the presence of this element in amounts above the permissible level (0.45 and 0.66 mg/kg, respectively).

The Pearson coefficient values listed in Table 5 (Cd and Cr were not considered) were calculated to identify the correlation between the elements in the studied fruit and vegetables.

A significant positive correlation was observed in the following metal pairs: Fe–Ni (r = 0.832), Fe–Zn (r = 0.896), and Ni–Zn (r = 0.841) at *p* < 0.01, Fe–Pb (r = 0.767) and Pb–Zn (r = 0.791) at *p* < 0.02, and Ni–Pb (r = 0.693) at *p* < 0.05. This means that Ni, Zn, and Pb concentrations may increase with increasing Fe content, Zn and Pb with increasing Ni content, and Zn with increasing Pb content, and vice versa, and indicates that these metals are mutually associated, commonly interact, and have a common source of pollution [50].

**Figure 4:**
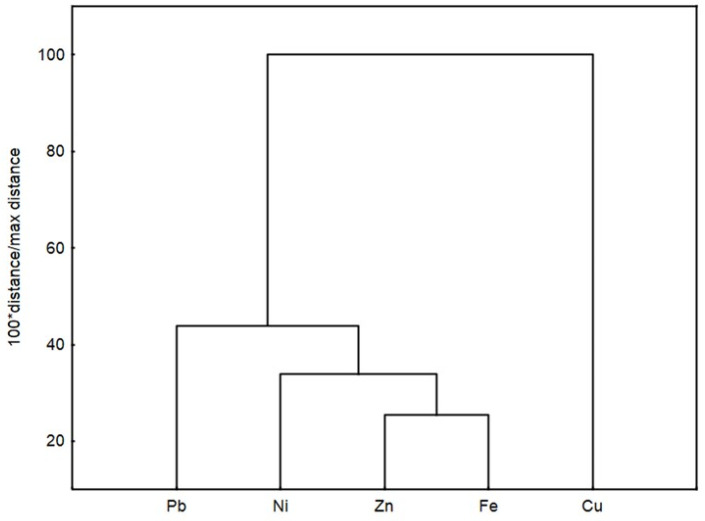
Dendrogram resulting from the hierarchical cluster analysis of the heavy metal concentration in the studied food.

The CA results presented as the dendrogram in Figure 4 show one main cluster with Pb, Ni, Zn, and Fe, indicating a good correlation between these metals, with a closer relation between Zn and Fe. Cu is outside this cluster and correlates poorly with the other metals.

In turn, comparing the amounts of metals in food and in the respective soils where it was cultivated, a significant correlation (*p* < 0.01) was found only in the case of iron (r = 0.833) and zinc (r = 0.823). For the other metal pairs, non-significant correlations were found, meaning that the content of one metal does not influence other metals’ contents in the studied soils and food. It suggests that the presence of these metals in food results from human activities carried out directly on the researched agricultural fields or in their surroundings.

### 2.4. Transfer Factor

**Figure 5:**
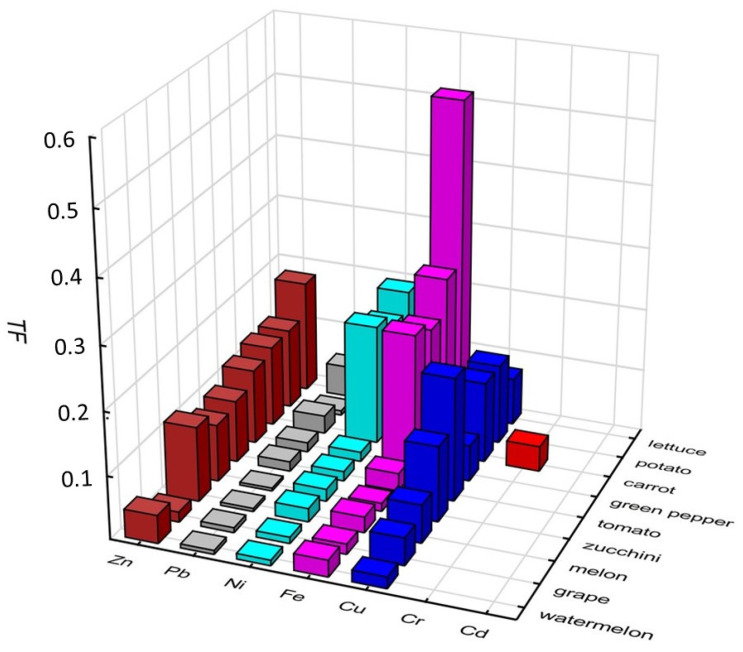
Transfer factors of the fruits and vegetables grown in the studied region of Boumerdes.

The heavy metal transfer from soil to plants is one of the main routes of human exposure to metals through the food chain. This phenomenon can be expressed numerically by calculating the transfer factor (TF) value from Equation (1). The TF is defined as the migration of metals from the soil to the edible part of the plants, making them available for consumption [51,52]. In this study, the TF was determined for the economically valuable parts of the plants. Several physicochemical factors of the soil and plant species govern this function. As can be seen from Figure 5, the TF values for fruits and vegetables grown at four studied sites varied in a wide range (0 and 0.040 for Cr; 0.018 and 0.196 for Cu; 0.013 and 0.515 for Fe; 0.009 to 0.193 for Ni; 0.005 to 0.050 for Pb; and 0.016 and 0.180 for Zn), depending on the type of plant and its place of growth. Cadmium was not transferred from the soil to the studied food.

Among the studied metals, the highest transfer of Cr to carrot, Cu to tomato, and Fe, Ni, Pb, and Zn to lettuce was observed. This variation can be explained by the differences in the concentrations of metals in the soils, the selective absorption of the elements by the plants [53], and the transmission in the individual plant tissues. The tested fruits absorbed significantly fewer metals than vegetables. The mean TF values for the studied vegetables decrease in the order Fe > Zn = Cu > Ni > Pb > Cr > Cd, similar to that obtained by Wang et al. [54] (except Cd). The relatively lower transfer factor values for Pb and Cr can be attributed to the stronger binding of these metals to the soil [47]. The results indicate that lettuce is the biggest accumulator of heavy metals, especially Fe, Ni, and Zn. On the other hand, the transfer factor values show that certain vegetables (e.g., zucchini) reduce the risk of human exposure to metal contamination in the soil [55].

### 2.5. Estimated Daily Intake of Heavy Metals

In being aware of the presence of heavy metals in the tested food and their adverse effects, it is necessary to determine the level of exposure of the body to these pollutants. The estimated daily intake (EDI) of the analyzed metals was calculated from Equation (2), according to the mean concentration of each metal in each food and the respective consumption rates. The EDI for individual food, total EDI for groups of the studied fruits and vegetables and all food, and maximum tolerable daily intake (MTDI) of the studied metals from the consumption of fruits and vegetables [56] are shown in Table 6. The highest total daily intake for all fruits was found for Zn (2.54·10^−3^ mg/day) and Cu (1.17·10^−3^ mg/day), with the highest EDI for melon (8.37·10^−4^ and 6.92·10^−4^ mg/day, respectively), whilst among the vegetables, the total daily intake was the highest for Fe (1.68·10^−2^ mg/day) and Zn (8.37·10^−3^ mg/day), with the highest EDI for potatoes (8.21·10^−3^ and 3.51·10^−3^ mg/day, respectively). In turn, the exposure to Cd and Cr was characterized by the lowest EDI values considering both the fruit alone, the vegetables alone, and their total. The total daily intakes of individual metals from all studied food were much lower than the MTDI values and constituted below 0.1% of this value for Cd, Cr, Cu, and Zn, 0.2% for Ni, and 0.6% for Pb.

### 2.6. Non-Carcinogenic Risk

**Figure 6:**
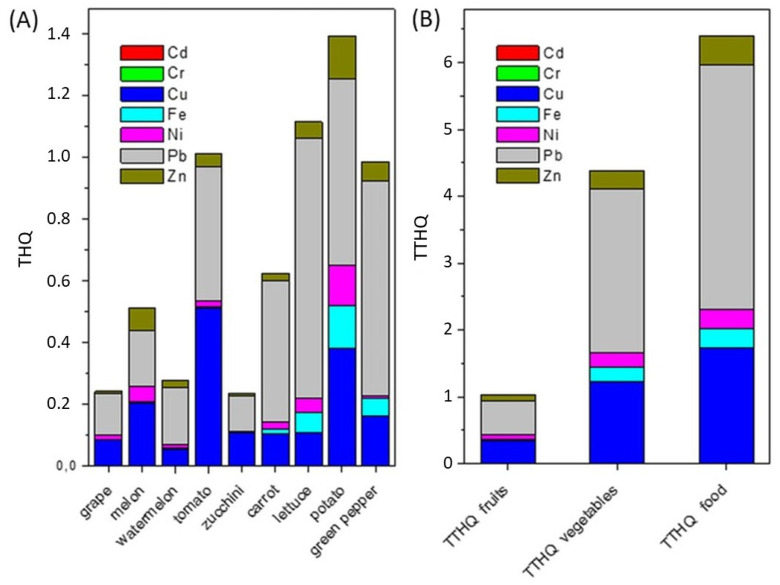
Target hazard quotient (THQ) (**A**) and total target hazard quotient (TTHQ) (**B**) for consumers of the food from the studied area.

The health risks caused by consuming contaminated fruits and vegetables were assessed based on the target hazard quotient (THQ) and total target hazard quotient (TTHQ) values, calculated from Equations (3) and (4). It is assumed that if the THQ is greater than 1, the exposed population is likely to suffer from the harmful effects of consuming contaminated food and appropriate interventions should be taken to eliminate the risk, and if the THQ < 1, the exposed population is unlikely to experience obvious adverse effects [57]. None of the tested fruits and vegetables had a THQ for single metals above the critical value of 1 and decreased (considering the highest values) from 0.834 to 0 in the following order: Pb > Cu > Zn = Fe > Ni > Cr > Cd (Figure 6A). The studied food can be considered potentially safe for health; however, attention should be paid to lettuce with a THQ value for Pb close to 1.

Regarding the TTHQ values for all the metals in individual food (Figure 6A), the following decreasing order was observed: potato (1.392) > lettuce (1.114) > tomato (1.013) > green pepper (0.985) > carrot (0.631) > melon (0.511) > watermelon (0.280) > grape (0.242). TTHQ values greater or close to 1 in potato, lettuce, tomato, and green pepper indicate a potential health risk when consumed continuously. If the consumption of only fruits is assumed (Figure 6B), the TTHQs of all studied metals are low (below 0.5), proving that the consumption of grapes, melons, and watermelons is relatively safe for human health. In turn, if the consumption of only vegetables is assumed, the TTHQ values of Pb and Cu exceed 1 (2.458 and 1.221, respectively), suggesting health risks to consumers from the contaminated vegetables. The overall potential non-carcinogenic effect of all elements and food simultaneously, according to Equation (5), is expressed by a hazard index (HI). Regarding the consumption of all studied fruits, the HI was 1.068, while for all studied vegetables, it was 4.471, which gave a combined HI of 6.524 for the consumption of all considered food. An HI in the range between 1 and 10 indicates that consumers may experience adverse health effects following the ingestion of the food [58], less from fruits than from vegetables.

### 2.7. Carcinogenic Risk

Among the studied heavy metals, cadmium and lead can cause both non-carcinogenic and carcinogenic effects, depending on the exposure dose. Since cadmium was identified at a concentration below the detection limit, the carcinogenic risk (CR) for the studied food was mainly derived from Pb intake. The CR for Pb was the highest in the case of carrot (7.78·10^−5^) and decreased as follows: lettuce (2.31·10^−5^) > green pepper (1.91·10^−5^) > zucchini (1.72·10^−5^) > tomato (1.48·10^−5^) > melon and watermelon (6.21·10^−6^) > grape (4.60·10^−6^) > potato (4.11·10^−6^). These values are below the acceptable limit value established by the USEPA to be 10^−4^, indicating no carcinogenic risk from consuming this metal in the studied fruits and vegetables.

## 3. Materials and Methods

### 3.1. The Study Area

**Figure 7:**
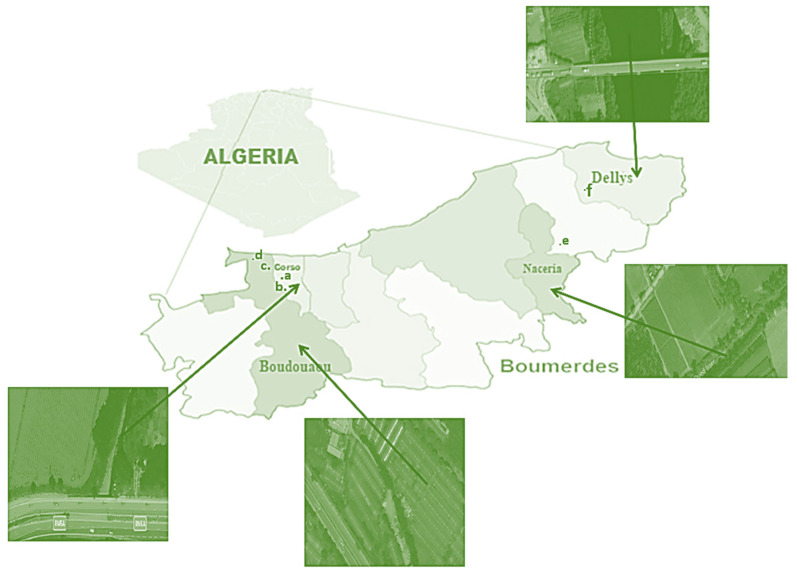
Study area with sampling sites and location of industrial activities (a—Ezmam/Solgen Paper Factory; b—Imotep Pharm; c—EURL Lepro Chemical Plant Pack; d—GC BFE; e—SNC Hassani; f—Socotid).

The present study was carried out from March to April 2020 in peri-urban areas of Boumerdes, located in the north of Algeria (Latitude: 36.7667, Longitude: 3.46667 36°46′0″ N, 3°28′0″ E and alt 2 m). As shown in Figure 7, the sampling sites were located along different rivers adjacent to road traffic in the regions of Boudouaou, Corso, Naceria, and Dellys.

The climate in Boumerdes is semi-arid, and rain in this area is much more important in the winter than in the summer. Boumerdes has an average annual temperature of 17.5 °C, with the highest temperature (about 27 °C) in August and the lowest (about 10 °C) in January. Over the year, the average rainfall is 672 mm, with about 97 mm in November and only 2 mm in July. In turn, the relative humidity of the year is the lowest in July (54.13%) and the highest in January (75.13%). The geological substrate of this region is regarded as a bilayer consisting of a Quaternary cover of mostly a Pliocene clayey sand layer and compact marl layer, with the exception of areas along rivers banks where additional Quaternary alluvium is present [59]. On the farms where the samples were taken, groundwater (in Boudouaou) or river water (in Corso, Naceria, and Dellys) is used to irrigate the crops. There are several industrial activities located in the region under study (Figure 7), e.g., paper production (a), pharmaceuticals (b), plastic packaging (c), food (e), engineering and procurement (d), and chemical wholesale (f).

### 3.2. Sample Preparation

Samples of water, soil, fruits, and vegetables collected from the selected farms were transported to the laboratory and properly prepared for the heavy metal analysis by the spectrometric method.

#### 3.2.1. Water Samples

Water samples (9 from each sampling site) were collected from the canals and river in 250 mL plastic bottles, as a mixture of two independent subsamples. After transporting to the laboratory, they were centrifuged to remove the suspended particles, filtered through a 0.45 µm PTFE membrane filter (Omnipore, Merck KGaA, Darmstadt, Germany), preserved with 1.0 mL of 70% HNO_3_, and then stored in a refrigerator to reduce volatilization and biodegradation between sampling and analysis.

#### 3.2.2. Soil Samples

Soil samples (9 from each sampling site) were collected randomly from agricultural plots using a stainless-steel auger at 0–30 cm depths and stored in plastic bags. From each plot, a soil sample of about 1 kg in weight consisting of a mixture of five subsamples collected from different sites of the studied area was taken. All samples were well mixed and riffled, and one-fourth of each sample was dried in the oven (Binder, Tuttlingen, Germany, model E028-230V-T) at 105 °C for 12 h. The dried samples were then ground and sieved with a 75 μm mesh size sieve and digested according to the method adopted by FAO/SIDA [60]. The soil pH was measured by a pH meter (Hanna Instruments HI 2211, Woonsocket, RI, USA) using a soil–water ratio of 1:1 (USEPA method 9045D, Washington, DC, USA).

A sample of 20 g of pulverized (75 μm) soil was weighed in a 400 mL tall-form beaker, and a mixture of 50 mL HCl and 20 mL HNO_3_ was slowly added to the sample. The contents of the beaker were continuously stirred with a glass rod to ensure that the soil sample was properly wetted and heated on a hot plate at 160 °C for a minimum of 45 min (until almost completely dry). Then, the sample was cooled, diluted in a 200 mL volumetric flask with deionized water, shaken and poured back into the beaker, and settled for 30 min. Finally, the sample was filtered and analyzed in 16 × 150 mm test tubes.

#### 3.2.3. Fruit and Vegetable Samples

Samples of fruits (30 from each of the 3 types), including *Vitis vinifera* (grape), *Cucumis melo var. saccharimus* (melon)*,* and *Citrullus vulgaris. Schrade* (watermelon), and vegetables (30 from each of the 6 types), including *Lycopersicum esculentum* L. (tomato)*, Cucurbita pepo* (zucchini)*, Daucus carota* (carrot)*, Lactuca sativa* (lettuce)*, Convolvulus Batatas* (potato)*,* and *Capsicum annuum* L. (green pepper), were collected from the study agricultural plots at different times depending on their availability. The samples were transported to the laboratory in paper bags, cleaned with deionized water to remove dust and extraneous matter, and dried in the oven at 105 °C for 12 h. In the case of plants with typically inedible peels, such as melon, watermelon, potato, and carrot, they were peeled before drying. The dried samples were then ground, homogenized, and stored in tightly closed, clean sample bottles until digestion. A sample of 2 g of fruit/vegetable (see Table S1) was weighed in a test tube and digested on a hot plate in three 30 min steps with (1) 10.0 mL of 30% H_2_O_2_, (2) 10.0 mL of 70% HNO_3_, and (3) 10.0 mL of HClO_4_. Then, the sample was cooled, carefully transferred into a 50 mL volumetric flask, rinsed, and diluted with deionized water. The prepared samples were placed in 16 × 150 mm test tubes and analyzed.

### 3.3. Chemicals

All chemical reagents used for the sample preparation were of analytical grade and purchased from MERCK Chemicals (Darmstadt, Germany). Quality control of the analytical step was carried out using reagent blanks (HNO_3_/H_2_SO_4_/HClO_4_ ratio 5:1:1) and NIST Standard Reference Materials (SRMs) 1643E (for water), 2709 (for soil), and 1570A (for vegetables). The relative standard deviation (RSD < 5%) was inspected by a tuning solution purchased from Thermo Fisher Scientific (Waltham, MA, USA). All samples and standards were diluted with deionized water.

### 3.4. Spectrometric Analysis

A flame atomic absorption spectrometer, Thermo Fisher Scientific ICE 3000 FAA (Waltham, MA, USA), fully automated and PC-controlled using SOLAAR Software 11.2 versions and equipped with a fast sequential operation for multi-element flame determinations, was used for Cd, Cr, Cu, Fe, Ni, Pb, and Zn analysis. The selected validation parameters of the applied method are shown in Table 7.

### 3.5. Calculations

For all calculations, the mean values of heavy metal concentrations in the water, soils, and food determined based on triplicate analysis of three independent samples were applied.

The transfer factor (TF) expressing the bioavailability of a metal at a particular position on a species of plant [61] was calculated based on the total metal content of the whole plant (edible part) without considering the various parts of the plant, using Equation (1) [51,52]:

(1) $$TF=\frac{C_{food}}{C_{soil}}$$

where C_food_—the mean metal concentration in fruit/vegetable (mg/kg); C_soil_—the mean metal concentration in soil (mg/kg).

The estimated daily intakes (EDIs) of the studied metals were calculated using Equation (2), taking into account the mean concentration of individual metals in food samples, the weight of fruits and vegetables consumed by an individual, and the average weight of an adult in Boumerdes equal to 63.6 kg:

(2) $$EDI=\frac{FIR\cdot C_{food}\cdot CF}{BW}$$

where FIR—the food ingestion rate (g/day/person); CF—the conversion factor equal to 0.085 (for fruits and vegetables); and BW—the body weight [62].

The non-cancerogenic risk associated with the consumption of heavy metals with food products can be expressed by the target hazard quotient (THQ) [51,63], total target hazard quotient (TTHQ), and hazard index (HI), calculated using Equations (3), (4), and (5), respectively.

(3) $$THQ=\frac{EFr\cdot ED\cdot FIR\cdot C_{food}}{RfD\cdot BW\cdot ATn\cdot 1000}$$

where EFr—frequency (365 days/year); ED—duration (77.8 years); RfD—the oral reference dose (mg/kg/day) equal to 0.001, 1.5, 0.04, 0.70, 0.02, 0.004, and 0.3 mg/kg/day, respectively, for Cd, Cr (it was assumed that all chromium ions in the studied food are in trivalent, non-carcinogenic form), Cu, Fe, Ni, Pb, and Zn; and ATn—the average time for non-carcinogens.

For the determination of the TTHQ, indicating the risk of exposing a person to all metals simultaneously, the sum of the THQ obtained for individual metals was applied [62]:

(4) $$TTHQ\left(individual\;food\right)=THQ\left(metal\;1\right)+THQ\left(metal\;2\right)+\ldots +THQ\left(metal\;n\right)$$

In order to assess the overall potential for non-carcinogenic effects from more than one food, a hazard index (HI) was formulated based on the USEPA guidelines for health risk assessment of chemical mixtures [64], as follows:

(5) $$HI=\sum_{i=1}^{n}{TTHQ}_{i}=TTHQ\left(food\;1\right)+TTHQ\left(food\;2\right)+\ldots +TTHQ\left(food\;n\right)$$

The carcinogenic risk can be expressed by the target cancer risk factor (TCR) determining the lifetime cancer risk, which can be calculated as follows [65]:

(6) $$TCR=\frac{EFr\cdot ED\cdot FIR\cdot C_{food}\cdot {CSF}_{o}}{BW\cdot ATc}$$

where CSF_o_– the cancer slope factor attributed to the carcinogenic metal equal to 6.3 and 8.5·10^−3^ (mg/kg/day)^−1^ for Cd and Pb [66,67], respectively; ATc—the average time for carcinogens.

### 3.6. Statistical Analysis

Statistica ver. 13.3 software (Tibco Software Inc., Palo Alto, CA, USA) was used for the statistical treatment of the data. The results of the metal concentration analysis and pH measurements are expressed as mean values ± standard deviation (SD). Specific relationships between the individual metal concentrations and pH values were determined by the Pearson correlation. The cluster analysis (CA) was applied to visualize the relationship between the measured parameters, the sampling site or the type of food cultivated. Analyses were performed on standardized data.

## 4. Conclusions

This study demonstrates that the concentrations of cadmium (Cd), copper (Cu), chromium (Cr), iron (Fe), nickel (Ni), lead (Pb), and zinc (Zn) in both the soils and irrigation waters of Boumerdes city are below the maximum permissible levels set by the FAO/WHO. The highest transfer factors (TFs) were observed for Cr in carrots, Cu in tomatoes, and Fe, Ni, Pb, and Zn in lettuce. The potential non-carcinogenic health risks, as assessed through the estimated daily intake (EDI) and target hazard quotient (THQ) values for individual heavy metals, are considered low. Similarly, the carcinogenic risk (CR) from consuming fruits and vegetables containing Pb is minimal. However, the total non-carcinogenic risk (TTHQ) associated with the consumption of potatoes, lettuce, tomatoes, and green peppers is near or exceeds a value of 1, indicating potential health impacts.

In conclusion, while heavy metal levels are within safety limits, this study underscores the necessity for the ongoing monitoring of heavy metal concentrations in both agricultural soils and crops. Ensuring crop safety requires the effective management of agricultural practices, such as avoiding the use of untreated wastewater or excessive artificial fertilizers and protecting the surrounding environment from industrial pollution.

## Figures and Tables

**Table 1:** The mean concentration of heavy metals in the irrigation water for crop production (SD: standard deviation; <LOD: below detection limit).

Sampling Site/Crop	C_water_ ± SD (mg/L)						
	Cd	Cr	Cu	Fe	Ni	Pb	Zn
Corso/ grape	<LOD	<LOD	0.82 ± 0.01	0.13 ± 0.02	0.11 ± 0.02	0.05 ± 0.01	0.05 ± 0.01
Corso/ melon	<LOD	<LOD	0.88 ± 0.01	0.11 ± 0.02	0.09 ± 0.01	0.03 ± 0.01	0.08 ± 0.01
Naceria/ watermelon	<LOD	<LOD	0.52 ±0.01	0.04 ± 0.01	0.14 ± 0.02	0.03 ± 0.01	0.13 ± 0.01
Boudouaou/ tomato	<LOD	0.09 ± 0.01	1.17 ± 0.02	0.67 ± 0.02	1.98 ± 0.04	0.09 ± 0.01	0.31 ± 0.02
Boudouaou/ zucchini	<LOD	<LOD	0.79 ± 0.02	0.54 ± 0.02	0.87 ± 0.02	0.07 ± 0.02	1.02 ± 0.04
Boudouaou/ carrot	<LOD	0.19 ± 0.01	0.31 ± 0.01	0.59 ± 0.01	0.11 ± 0.02	0.07 ± 0.02	0.47 ± 0.02
Dellys/lettuce	<LOD	0.11 ± 0.02	0.70 ± 0.03	1.10 ± 0.02	0.02 ± 0.01	0.82 ± 0.05	0.62 ± 0.01
Dellys/potato	<LOD	0.09 ± 0.01	0.56 ± 0.01	0.97 ± 0.04	0.91 ± 0.03	0.42 ± 0.03	0.88 ± 0.04
Dellys/green pepper	<LOD	0.26 ± 0.01	0.87 ± 0.02	0.87 ± 0.03	0.02 ± 0.01	0.04 ± 0.01	0.54 ± 0.02
ML (mg/L) [24,25]	0.01	0.10	0.20	5.00	0.20	5.00	2.00

**Table 2:** pH and mean concentration of heavy metals in the studied soils (SD: standard deviation; <LOD: below detection limit).

Sampling Site/Crop	pH		C_soil_ ± SD (mg/kg)						
			Cd	Cr	Cu	Fe	Ni	Pb	Zn
Corso/ grape	6.99 ± 0.03		0.09 ± 0.02	12.17 ± 0.85	57.12 ± 6.28	53.21 ± 8.47	19.52 ±1.85	61.11 ±3.24	76.12 ± 6.43
Corso/ melon	7.13 ± 0.05		0.07 ± 0.02	11.39 ± 0.35	63.23 ± 9.56	57.64 ± 7.65	21.24 ± 2.27	54.22 ± 5.68	83.27 ± 8.67
Naceria/ watermelon	6.80 ± 0.08		0.02 ± 0.00	6.91 ± 0.60	52.10 ± 4.71	42.74 ± 7.85	13.55 ± 3.11	49.22 ± 2.29	70.12 ± 4.78
Boudouaou/ tomato	7.11 ± 0.07		0.04 ± 0.00	11.20 ± 0.50	66.25 ± 1.05	62.33 ± 1.02	15.60 ± 2.28	72.35 ± 1.20	81.24 ± 1.50
Boudouaou/ zucchini	6.57 ± 0.07		0.02 ± 0.01	8.92 ± 0.29	78.00 ± 1.32	72.97 ± 1.04	10.82 ± 4.34	92.82 ± 1.63	71.9 ± 5.32
Boudouaou/ carrot	6.92 ± 0.09		<LOD	2.24 ± 0.80	52.64 ± 6.51	89.11 ± 10.48	3.25 ± 0.89	101.20 ± 8.43	95.12 ± 1.33
Dellys/ lettuce	6.32 ± 0.13		<LOD	2.31 ± 0.20	74.64 ± 8.68	105.35 ± 1.63	5.23 ± 1.87	68.90 ± 1.31	108.21 ± 1.87
Dellys/ potato	7.13 ± 0.08		<LOD	3.50 ± 0.11	38.25 ± 1.34	120.42 ± 10.95	4.94 ± 0.85	110.25 ± 10.84	102.32 ± 1.89
Dellys/ green pepper	7.31 ± 0.09		<LOD	2.61 ± 0.2	60.20 ± 10.57	95.43 ± 1.74	7.27 ± 0.35	79.52 ± 1.11	82.40 ± 1.05
ML (mg/kg) [35,36]		-	3	150	140	5000	75	300	300

**Table 3:** Pearson correlation coefficients of the heavy metal pollutants in the studied soils (significant values are bolded; * *p* < 0.05; ** *p* < 0.01).

Metal	pH_soil_	Cd	Cr	Cu	Fe	Ni	Pb	Zn
pH_soil_	1							
Cd	0.244	1						
Cr	0.169	0.880 **	1					
Cu	−0.559	0.090	0.250	1				
Fe	−0.056	−0.680 *	−0.784 *	−0.197	1			
Ni	0.250	0.916 **	0.939 **	0.182	−0.827 **	1		
Pb	0.073	−0.578	−0.523	−0.277	0.750 *	−0.730 *	1	
Zn	−0.199	−0.459	−0.658	−0.180	0.833 **	−0.644	**0.456**	1

**Table 4:** The mean concentration of heavy metals in the studied fruits and vegetables (SD: standard deviation; <LOD: below detection limit; n.a.: not available).

Food	C_food_ ± SD (mg/kg)						
	Cd	Cr	Cu	Fe	Ni	Pb	Zn
Fruits							
Grape	<LOD	<LOD	2.74 ± 0.16	0.94 ± 0.02	0.21 ± 0.01	0.43 ± 0.01	1.33 ± 0.12
Melon	<LOD	<LOD	3.45 ± 0.05	1.32 ± 0.02	0.42 ± 0.03	0.31 ± 0.06	9.01 ± 0.03
Watermelon	<LOD	<LOD	0.94 ± 0.02	1.12 ± 0.05	0.12 ± 0.02	0.31 ± 0.02	3.12 ± 0.06
ML [44]	0.05	1.0	4.5	n.a.	0.8	0.1	n.a.
Vegetables							
Tomato	<LOD	<LOD	13.01 ± 0.45	1.75 ± 0.05	0.24 ± 0.05	1.11 ± 0.04	8.04 ± 0.52
Zucchini	<LOD	<LOD	9.36 ± 0.82	0.94 ± 0.02	< LOD	0.43 ± 0.01	6.54 ± 0.51
Carrot	<LOD	0.09 ± 0.01	6.75 ± 0.29	17.87 ± 0.22	0.62 ± 0.02	2.91 ± 0.10	12.21 ± 0.41
Potato	<LOD	<LOD	4.86 ± 0.13	30.72 ± 0.16	0.82 ± 0.02	0.77 ± 0.02	13.14 ± 0.13
Lettuce	<LOD	<LOD	5.62 ± 0.26	54.20 ± 0.64	1.01 ± 0.19	3.45 ± 0.26	19.52 ± 0.67
Green pepper	<LOD	<LOD	3.44 ± 0.15	21.00 ± 0.88	0.11 ± 0.01	1.19 ± 0.08	9.84 ± 0.18
ML [44]	0.05	1.0	40	425	10	0.1	100

**Table 5:** Pearson correlations of the heavy metal pollutants in the studied food (significant values are bolded; * *p* < 0.05; ** *p* < 0.02; *** *p* < 0.01).

Metal	Cu	Fe	Ni	Pb	Zn
Cu	1				
Fe	−0.068	1			
Ni	−0.023	0.832 ***	1		
Pb	0.203	0.767 **	0.693 *	1	
Zn	0.197	0.896 ***	0.841 ***	0.791 **	1

**Table 6:** Estimated daily intake (EDI) of heavy metals with corresponding maximum tolerable daily intake (MTDI) for the Boumerdes population (n.a.—not available).

Food	FIR (g/Day/Person)		EDI (mg/Day)						
			Cd	Cr	Cu	Fe	Ni	Pb	Zn
Fruits									
Grape		80	<1.39 × 10^−6^	<8.02 × 10^−6^	2.93 × 10^−4^	1.01 × 10^−4^	2.25 × 10^−5^	4.60 × 10^−5^	1.09 × 10^−4^
Melon		150	<2.61 × 10^−6^	<1.50 × 10^−5^	6.92 × 10^−4^	2.65 × 10^−4^	8.42 × 10^−5^	6.21 × 10^−5^	1.81 × 10^−3^
Watermelon		150	<2.61 × 10^−6^	<1.50 × 10^−5^	1.88 × 10^−4^	2.25 × 10^−4^	2.41 × 10^−5^	6.21 × 10^−5^	6.25 × 10^−3^
All fruits			<6.60 × 10^−6^	<3.81 × 10^−5^	1.17 × 10^−3^	5.91 × 10^−4^	1.31 × 10^−4^	1.70 × 10^−4^	2.54 × 10^−3^
Vegetables									
Tomato	100		<1.74 × 10^−6^	<1.00 × 10^−5^	1.74 × 10^−4^	2.34 × 10^−4^	3.21 × 10^−5^	1.48 × 10^−4^	1.07 × 10^−3^
Zucchini	30		<5.21 × 10^−7^	<3.01 × 10^−6^	3.75 × 10^−4^	6.70 × 10^−4^	<1.00 × 10^−6^	3.93 × 10^−5^	2.62 × 10^−4^
Carrot	40		<6.95 × 10^−7^	4.01 × 10^−6^	3.61 × 10^−4^	9.55 × 10^−4^	3.31 × 10^−5^	1.56 × 10^−4^	6.53 × 10^−4^
Potato	200		<3.47 × 10^−6^	<2.00 × 10^−5^	1.30 × 10^−3^	8.21 × 10^−4^	2.19 × 10^−4^	2.06 × 10^−4^	3.51 × 10^−3^
Lettuce	50		<8.69 × 10^−7^	<5.01 × 10^−6^	3.76 × 10^−4^	3.89 × 10^−3^	7.42 × 10^−5^	2.87 × 10^−4^	1.30 × 10^−3^
Green pepper	120		<2.08 × 10^−6^	<1.20 × 10^−5^	5.52 × 10^−4^	3.40 × 10^−3^	1.76 × 10^−5^	2.36 × 10^−4^	1.57 × 10^−3^
All vegetables			<9.38 × 10^−6^	<5.41 × 10^−5^	3.14 × 10^−3^	1.68 × 10^−2^	3.76 × 10^−5^	1.07 × 10^−3^	8.37 × 10^−3^
All food			<1.60 × 10^−5^	<9.22 × 10^−5^	4.31 × 10^−3^	1.73 × 10^−2^	5.07 × 10^−4^	1.24 × 10^−3^	1.09 × 10^−2^
MTDI [56]			0.021	0.2	30	n.a.	0.3	0.21	60

**Table 7:** Selected validation parameters of the method used.

Element	λ [nm]	Concentration Range [mg/L]	Correlation Coefficient (R^2^)	Limit of Detection		
				For Water [mg/L]	For Soil [mg/kg]	For Food [mg/kg]
Cd	228.8	0–2.0	0.9990	0.0005	0.005	0.013
Cr	357.9	0–2.0	0.9982	0.003	0.03	0.075
Cu	324.8	0–2.0	0.9980	0.001	0.01	0.025
Fe	248.3	0–2.0	0.9984	0.002	0.02	0.050
Ni	232.0	0–2.0	0.9974	0.001	0.01	0.025
Pb	217.0	0–2.0	0.9996	0.006	0.06	0.150
Zn	213.9	0–20.0	0.9950	0.004	0.04	0.100

## Data Availability

Data are contained within the article and Supplementary Materials.

## Supplementary Materials

The following supporting information can be downloaded at: https://www.mdpi.com/article/10.3390/molecules29174187/s1, Table S1: Detailed data for the analyzed fruit and vegetable samples.

## References

[1] Rehman Z.U., Khan S., Shah M.T., Brusseau M.L., Khan S.A., Mainhagu J. (2018). Transfer of Heavy Metals from Soils to Vegetables and Associated Human Health Risks at Selected Sites in Pakistan. Pedosphere.

[2] Rabadjieva D., Tepavitcharova S., Kovacheva A., Gergulova R., Ilieva R., Vladov I., Nanev V., Gabrashanska M., Karavoltsos S. (2021). Trace Metals Accumulation in the Eco-System Water-Soil-Vegetation (*Agropyron cristatum*)-Common Voles (*Microtus arvalis*)-Parasites (*Hymenolepis diminuta*) in Radnevo Region, Bulgaria. J. Trace Elem. Med. Biol..

[3] Guerrieri N., Mazzini S., Borgonovo G. (2024). Food Plants and Environmental Contamination: An Update. Toxics.

[4] Saleem M., Pierce D., Wang Y., Sens D.A., Somji S., Garrett S.H. (2024). Heavy Metal(Oid)s Contamination and Potential Ecological Risk Assessment in Agricultural Soils. J. Xenobiot..

[5] Duruibe J.O., Ogwuegbu M.O.C., Egwurugwu J.N. (2007). Heavy Metal Pollution and Human Biotoxic Effects. Int. J. Phys. Sci..

[6] Osae R., Nukpezah D., Amoako Darko D., Mensah A. (2023). Heavy Metal Mobility, Bioavailability, and Potential Toxicity in Sediments of the Korle Lagoon in Ghana. Int. J. Environ. Stud..

[7] Djarmouni M., Sekia I., Ameni D., Ikessoulen T., Baghiani A. (2023). Impact of Toxic Heavy Metals and Their Concentration in Zygophyllum Species, *Mentha longifolia*, and *Thymus vulgaris* Traditional Medicinal Plants Consumed in Setif-Algeria. Eur. J. Biol..

[8] Briffa J., Sinagra E., Blundell R. (2020). Heavy Metal Pollution in the Environment and Their Toxicological Effects on Humans. Heliyon.

[9] Milanković V., Tasić T., Leskovac A., Petrović S., Mitić M., Lazarević-Pašti T., Novković M., Potkonjak N. (2024). Metals on the Menu—Analyzing the Presence, Importance, and Consequences. Foods.

[10] Genchi G., Sinicropi M.S., Lauria G., Carocci A., Catalano A. (2020). The Effects of Cadmium Toxicity. Int. J. Environ. Res. Public Health.

[11] Yu G., Zheng W., Wang W., Dai F., Zhang Z., Yuan Y., Wang Q. (2017). Health Risk Assessment of Chinese Consumers to Cadmium via Dietary Intake. J. Trace Elem. Med. Biol..

[12] Wang G., Su M.Y., Chen Y.H., Lin F.F., Luo D., Gao S.F. (2006). Transfer Characteristics of Cadmium and Lead from Soil to the Edible Parts of Six Vegetable Species in Southeastern China. Environ. Pollut..

[13] Steffan J.J., Brevik E.C., Burgess L.C., Cerdà A. (2018). The Effect of Soil on Human Health: An Overview. Eur. J. Soil Sci..

[14] Rerknimitr P., Kantikosum K., Chottawornsak N., Tangkijngamvong N., Kerr S.J., Prueksapanich P., Sithisarankul P., Kumtornrut C., Asawanonda P., Sutheparuk S. (2019). Chronic Occupational Exposure to Lead Leads to Significant Mucocutaneous Changes in Lead Factory Workers. J. Eur. Acad. Dermatol. Venereol..

[15] Huang J.L., Mo Z.Y., Li Z.Y., Liang G.Y., Liu H.L., Aschner M., Ou S.Y., Zhou B., Chen Z.M., Jiang Y.M. (2021). Association of Lead and Cadmium Exposure with Kidney Stone Incidence: A Study on the Non-Occupational Population in Nandan of China. J. Trace Elem. Med. Biol..

[16] Kawatra B.L., Bakhetia P. (2008). Consumption of Heavy Metal and Minerals by Adult Women through Food in Sewage and Tube-Well Irrigated Area around Ludhiana City (Punjab, India). J. Hum. Ecol..

[17] Aftab K., Iqbal S., Khan M.R., Busquets R., Noreen R., Ahmad N., Kazimi S.G.T., Karami A.M., Al Suliman N.M.S., Ouladsmane M. (2023). Wastewater-Irrigated Vegetables Are a Significant Source of Heavy Metal Contaminants: Toxicity and Health Risks. Molecules.

[18] Fytianos K., Katsianis G., Triantafyllou P., Zachariadis G. (2001). Accumulation of Heavy Metals in Vegetables Grown in an Industrial Area in Relation to Soil. Bull. Environ. Contam. Toxicol..

[19] Sharma R.K., Agrawal M., Marshall F.M. (2008). Atmospheric Deposition of Heavy Metals (Cu, Zn, Cd and Pb) in Varanasi City, India. Environ. Monit. Assess..

[20] Kachenko A.G., Singh B. (2006). Heavy Metals Contamination In Vegetables Grown In Urban And Metal Smelter Contaminated Sites In Australia. Water Air Soil Pollut..

[21] Cherfouh R., Lucas Y., Derridj A., Merdy P. (2018). Long-Term, Low Technicality Sewage Sludge Amendment and Irrigation with Treated Wastewater under Mediterranean Climate: Impact on Agronomical Soil Quality. Environ. Sci. Pollut. Res..

[22] Baziz D.A., Maazouzi A., Lachache S. (2022). Physical-Chemical Characterisation of the Urban Wastewater—Case Study of the Boumerdes Region, North-Algeria. J. Water Land Dev..

[23] Ghemmit-Doulache N. (2018). Heavy Metals Detection in Soil Irrigated by STEP of Boumerdes-Algeria. J. Res. Green Chem..

[24] Drechsel P., Marjani Zadeh S., Pedrero F. (2023). Water Quality in Agriculture: Risks and Risk Mitigation.

[25] USEPA (United States Environmental Protection Agency) (2012). Guidelines for Water Reuse 600/R-12/618.

[26] Khadidja B., Nadjiba C. (2020). Biochemical Approach to Assess Groundwater Pollution by Heavy Metals Pollutants and Organics (Case Reghaia Lake, Algeria). Highlights Bioinform..

[27] Belkhiri L., Tiri A., Mouni L. (2018). Assessment of Heavy Metals Contamination in Groundwater: A Case Study of the South of Setif Area, East Algeria. Achievements and Challenges of Integrated River Basin Management.

[28] Boukhalfa C. (2007). Heavy Metals in the Water and Sediments of Oued Es-Souk, Algeria, a River Receiving Acid Effluents from an Abandoned Mine. Afr. J. Aquat. Sci..

[29] Bouragba S., Komai K., Nakayama K. (2019). Assessment of Distributed Hydrological Model Performance for Simulation of Multi-Heavy-Metal Transport in Harrach River, Algeria. Water Sci. Technol..

[30] Chaouch N., Birech S., Messaoudi I. (2019). Detection of Toxic Heavy Metals in the Water of Chott Ain El Beida (Bowl of Ouargla, South-East of Algeria). Mater. Biomater. Sci..

[31] Guezgouz N., Parisi C., Boubsil S., Grieco G., Hana S.A., Guerriero G. (2021). Heavy Metals Assessment in the Medjerda River Basin (Northeastern Algeria): A Preliminary Water Analysis and Toad Skin Biopsy. Proc. Zool. Soc..

[32] Krika A. (2018). Assessment of Heavy Metals Pollution in Water and Sediments of Djendjen River, North Eastern Algeria. Pollution.

[33] Kaddour K., Smail M. (2017). Assessment of Heavy Metal Pollution Due to the Lead—Zinc Mine at the Ain Azel Area (Northeast of Algeria). J. Environ. Res..

[34] Kicińska A., Pomykała R., Izquierdo-Diaz M. (2022). Changes in Soil PH and Mobility of Heavy Metals in Contaminated Soils. Eur. J. Soil Sci..

[35] Saha J.K., Selladurai R., Coumar M.V., Dotaniya M.L., Kundu S., Patra A.K. (2017). Environmental Chemistry for a Sustainable World, Vol.10: Soil Pollution—An Emerging to Agriculture.

[36] (2018). State Administration of Market Regulation Soil Environmental Quality—Risk Control Standard for Soil of Agricultural Land.

[37] Singh S., Kumar M. (2006). Heavy Metal Load of Soil, Water and Vegetables in Peri-Urban Delhi. Environ. Monit. Assess..

[38] Laribi A., Shand C., Wendler R., Mouhouche B., Colinet G. (2019). Concentrations and Sources of Cd, Cr, Cu, Fe, Ni, Pb and Zn in Soil of the Mitidja Plain, Algeria. Toxicol. Environ. Chem..

[39] Maas S., Scheifler R., Benslama M., Crini N., Lucot E., Brahmia Z., Benyacoub S., Giraudoux P. (2010). Spatial Distribution of Heavy Metal Concentrations in Urban, Suburban and Agricultural Soils in a Mediterranean City of Algeria. Environ. Pollut..

[40] El-Hadef El-Okki M., Sahli L., Bentellis A., Azzoug R., Laing G.D., Rached O. (2016). Assessment of Metal Contamination in Soil Banks of the Rhumel Wadi (Northeast Algeria). Toxicol. Environ. Chem..

[41] Sellami S., Zeghouan O., Dhahri F., Mechi L., Moussaoui Y., Kebabi B. (2022). Assessment of Heavy Metal Pollution in Urban and Peri-Urban Soil of Setif City (High Plains, Eastern Algeria). Environ. Monit. Assess..

[42] Benhaddya M.L., Hadjel M. (2014). Spatial Distribution and Contamination Assessment of Heavy Metals in Surface Soils of Hassi Messaoud, Algeria. Environ. Earth Sci..

[43] Ramdani S., Amar A., Belhsaien K., El Hajjaji S., Ghalem S., Zouahri A., Douaik A. (2018). Assessment of Heavy Metal Pollution and Ecological Risk of Roadside Soils in Tlemcen (Algeria) Using Flame-Atomic Absorption Spectrometry. Anal. Lett..

[44] (2001). Food Additives and Contaminants. Codex Alimentarius Commission. Joint FAO/WHO Food Standards Program.

[45] Guerra F., Trevizam A.R., Muraoka T., Marcante N.C., Canniatti-Brazaca S.G. (2012). Heavy Metals in Food Chain Heavy Metals in Vegetables and Potential Risk for Human Health. Sci. Agric..

[46] Rutigliano F.A., Marzaioli R., De Crescenzo S., Trifuoggi M. (2019). Human Health Risk from Consumption of Two Common Crops Grown in Polluted Soils. Sci. Total Environ..

[47] Bounar A., Boukaka K., Leghouchi E. (2020). Determination of Heavy Metals in Tomatoes Cultivated under Green Houses and Human Health Risk Assessment. Qual. Assur. Saf. Crops Foods.

[48] de Sousa F.F., do Carmo M.G.F., Lima E.S.A., da Costa Barros de Souza C., do Amaral Sobrinho N.M.B. (2020). Lead and Cadmium Transfer Factors and the Contamination of Tomato Fruits (*Solanum lycopersicum*) in a Tropical Mountain Agroecosystem. Bull. Environ. Contam. Toxicol..

[49] Grochowska-Niedworok E., Nieć J., Baranowska R. (2020). Assessment of Cadmium and Lead Content in Tomatoes and Tomato Products. Rocz. Panstw. Zakl. Hig. Ann. Natl. Inst. Hyg..

[50] Ametepey S.T., Cobbina S.J., Akpabey F.J., Duwiejuah A.B., Abuntori Z.N. (2018). Health Risk Assessment and Heavy Metal Contamination Levels in Vegetables from Tamale Metropolis, Ghana. Int. J. Food Contam..

[51] Cheshmazar E., Arfaeinia H., Karimyan K., Sharafi H., Hashemi S.E. (2018). Dataset for Effect Comparison of Irrigation by Wastewater and Ground Water on Amount of Heavy Metals in Soil and Vegetables: Accumulation, Transfer Factor and Health Risk Assessment. Data Brief.

[52] Thien B.N., Ba V.N., Man M.T., Hong Loan T.T. (2021). Analysis of the Soil to Food Crops Transfer Factor and Risk Assessment of Multi-Elements at the Suburban Area of Ho Chi Minh City, Vietnam Using Instrumental Neutron Activation Analysis (INAA). J. Environ. Manag..

[53] Zheng N., Wang Q., Zhang X., Zheng D., Zhang Z., Zhang S. (2007). Population Health Risk Due to Dietary Intake of Heavy Metals in the Industrial Area of Huludao City, China. Sci. Total Environ..

[54] Wang Y., Qiao M., Liu Y., Zhu Y. (2012). Health Risk Assessment of Heavy Metals in Soils and Vegetables from Wastewater Irrigated Area, Beijing-Tianjin City Cluster, China. J. Environ. Sci..

[55] Mapanda F., Mangwayana E.N., Nyamangara J., Giller K.E. (2007). Uptake of Heavy Metals by Vegetables Irrigated Using Wastewater and the Subsequent Risks in Harare, Zimbabwe. Phys. Chem. Earth.

[56] Shaheen N., Irfan N.M., Khan I.N., Islam S., Islam M.S., Ahmed M.K. (2016). Presence of Heavy Metals in Fruits and Vegetables: Health Risk Implications in Bangladesh. Chemosphere.

[57] Chowdhury A.I., Shill L.C., Raihan M.M., Rashid R., Bhuiyan M.N.H., Reza S., Alam M.R. (2024). Human Health Risk Assessment of Heavy Metals in Vegetables of Bangladesh. Sci. Rep..

[58] Singh A., Sharma R.K., Agrawal M., Marshall F.M. (2010). Health Risk Assessment of Heavy Metals via Dietary Intake of Foodstuffs from the Wastewater Irrigated Site of a Dry Tropical Area of India. Food Chem. Toxicol..

[59] Moulouel H., Bensalem R., Machane D., Bendaoud A., Gharbi S., Oubaiche E.H., Ousalem H., Skendri W. (2017). High Resistant Sand Injected Marl and Low Resistant Damaged Marl to Locate and Characterize the Thénia Fault Zone in Boumerdes City (North-Central Algeria). Pure Appl. Geophys..

[60] FAO (Food and Agriculture Organization) (1983). Manual of Methods in Aquatic Environment Research. Part 9. Analysis of Metals and Organochlorine in Fish. FAO Fish Technical Paper 212. Section 2.

[61] Eliku T., Leta S. (2017). Heavy Metals Bioconcentration from Soil to Vegetables and Appraisal of Health Risk in Koka and Wonji Farms, Ethiopia. Environ. Sci. Pollut. Res..

[62] Yaqub G., Khan A., Zishan Ahmad M., Irshad U. (2021). Determination of Concentration of Heavy Metals in Fruits, Vegetables, Groundwater, and Soil Samples of the Cement Industry and Nearby Communities and Assessment of Associated Health Risks. J. Food Qual..

[63] Njuguna S.M., Makokha V.A., Yan X., Gituru R.W., Wang Q., Wang J. (2019). Health Risk Assessment by Consumption of Vegetables Irrigated with Reclaimed Waste Water: A Case Study in Thika (Kenya). J. Environ. Manag..

[64] USEPA (United States Environmental Protection Agency) (2000). Supplementary Guidance for Conducting Health Risk Assessment of Chemical Mixtures Risk Assessment Forum Technical Panel.

[65] Javed M., Usmani N. (2016). Accumulation of Heavy Metals and Human Health Risk Assessment via the Consumption of Freshwater Fish *Mastacembelus Armatus* Inhabiting, Thermal Power Plant Effluent Loaded Canal. SpringerPlus.

[66] USEPA (United State Environmental Protection Agency) (2024). Risk Based Screening Table. Composite Tables.

[67] Battsengel E., Murayama T., Fukushi K., Nishikizawa S., Chonokhuu S., Ochir A., Tsetsgee S., Davaasuren D. (2020). Ecological and Human Health Risk Assessment of Heavy Metal Pollution in the Soil of the Ger District in Ulaanbaatar, Mongolia. Int. J. Environ. Res. Public Health.

