# Supplementary material for: Presence of Heavy Metals in Irrigation Water, Soils, Fruits, and Vegetables: Health Risk Assessment in Peri-Urban Boumerdes City, Algeria
Source: Molecules. 2024 Sep 4;29(17):4187. doi: 10.3390/molecules29174187 (PMC11397094; doi:10.3390/molecules29174187)
Supplement: Supplementary file 1 [file molecules-29-04187-s001.zip › molecules-3165535-supplementary.pdf]

Presence Of Heavy Metals In Irrigation Water, Soils, Fruits And Vegetables: Health Risk Assessment In Peri-Urban Boumerdes City, Algeria

Mohamed Younes Aksouh, Naima Boudieb, Nadjib Benosmane, Yacine Moussaoui, Rajmund Michalski, Justyna Klyta, Joanna Kończyk

Table S1. Detailed data for the analyzed fruit and vegetable samples

**Sample**	**Latin name**	**Variety**	**Analyzed part**
Grape	*Vitis vinifera*	Red globe	Fruits with peel
Melon	*Cucumis melo*	saccharimus	Fruits without peel
Watermelon	*Citrullus vulgaris. Schrade*	Sugar Baby	Fruits without peel
Tomato	*Lycopersicum esculentum L.*	Marmande VR	Fruits with peel
Zucchini	*Cucurbita pepo*	Green zucchini from Algiers	Fruits with peel
Carrot	*Daucus carota*	Muscade d'Alger	Root without peel
Potato	*Convolvulus Batatas*	Yellow potato from Malaga	Tuber without peel
Lettuce	*Lactuca sativa*	Scarlina	Leaves
Green pepper	*Capsicum annuum L.*	Doux d'Espagne	Fruits
